# Learner Analysis to Inform the Design and Development of a Serious Game for Nongaming Female Emerging Health Care Preprofessionals: Qualitative Sample Study

**DOI:** 10.2196/16003

**Published:** 2020-02-06

**Authors:** Kevin Glover, Alec Bodzin

**Affiliations:** 1 Lehigh University College of Education Bethlehem, PA United States

**Keywords:** games, health care, education, females, motivation, instructional design

## Abstract

**Background:**

Overall, 75% of health care practitioners are women, but half of all females do not play digital games of any kind. There is no consensus in the literature regarding optimal design elements to maximize the efficacy of serious games. To capitalize on the promise of serious games in health care education, it is important for instructional designers to understand the underlying learners’ values, attitudes, and beliefs that might motivate nongaming female health care preprofessional students to independently choose to persistently play serious games to mastery.

**Objective:**

Specifically, the aim of this study was to seek answers to 2 questions. First, what values, attitudes, and beliefs contribute to the nongaming behaviors of 12th-grade female emerging health care preprofessionals? Second, how do the values, attitudes, and beliefs of 12th-grade female emerging health care preprofessionals align with important design features of serious games?

**Methods:**

In this study, a learner analysis was conducted using semistructured interviews with 8 12th-grade college-bound female health science students to better understand learners’ values, attitudes, and beliefs to inform the design and development of a serious game. These interviewees represented a diverse subset of the female emerging health care preprofessionals who self-identified themselves as not playing games at all, not very often, or infrequently.

**Results:**

The findings suggest that the study participants exhibited a complex fusion of desire for both accomplishment and affiliation. The participants were all independent, competitive, and prosocial leaders. They thought strategically and consciously self-limited their leisure time to achieve personally meaningful long-term goals. They embraced overcoming expected failures and aimed to achieve relevant high-stakes wins in all academic, athletic, extracurricular, and leisure activities they valued while consciously avoiding what they considered to be non–goal-oriented activities.

**Conclusions:**

The results of this study reinforce the need for a robust learner analysis to identify the multifaceted behavioral characteristics of targeted learners before the design and development of serious games. The common characteristics of the 12th-grade female health science students in this study suggest that they will choose to invest their limited leisure time playing a personally meaningful, preprofessionally authentic serious game if the collective design elements are aligned with the students’ self-conceptualization of their present or future selves.

## Introduction

On the basis of the existing gender-based gaming research, half of all females do not play digital games of any kind [1-7]. This generalized finding is consistent with the digital game play consumption of female health science students. Kron et al reported that female medical students (N=115) played video games rarely (66%) or infrequently (22%), although 97% considered themselves to be basic to intermediate computer users [8]. More recently, the authors described similar results for a population of 17- and 18-year-old 12th-grade health science students (N=44) in which 56.7% of females (n=37) and 14.8% of males (n=7) reported that they did not play games at all or did not play very often. Notably, these female and male students reported that they used computers 86% and 57.1%, respectively, every day [9]. These findings are inconsistent with those of other researchers who suggest that young females who do not play digital games are somehow technologically disadvantaged [3,10]. All the young women (n=37) and men (n=7) in the authors’ study were college-bound science, technology, engineering, and mathematics (STEM) oriented students [9]. The number of females (84.1%), compared with males (15.9%), in this 12th-grade elective health science class was also consistent with the growing number of women in the health professions [9]. Overall, 75% of health care practitioners are women (N=9,420,000) [11]. Over 40% of physicians and surgeons now in the United States are females. Health care occupations, including pharmacists (63.4%), nurse practitioners (87.2%), physician assistants (72%), occupational therapists (86.8%), and physical therapists (69.5%), are primarily populated by women [11].

A robust targeted learner analysis should precede the design and development of any serious simulation-based or game-based educational intervention, but such analysis rarely occurs or is rarely reported [5,12-17]. The learner analysis described in this study was conducted to better understand the values, attitudes, and beliefs of 12th-grade female college-bound health science students before developing a serious simulation-based game for this population of students.

### Background

The use of simulation in clinical education has been associated with positive results in the acquisition of knowledge, confidence, skills, attitudes, and behaviors for nearly 6 decades in modern health care contexts [18]. Simulation-based education provides opportunities for standardized, deliberate practice, with feedback for the correction of errors until reconstruction and encoding of new understandings are achieved. The use of simulation in clinical education approximates a dose-response relationship in which more practice results in increased knowledge, improved confidence, and improved skills [19]. Although the published dose-response results of learning outcomes in non–intervention-simulated settings are abundant and statistically impressive [20], research regarding the transfer and impact of new knowledge and skills gained in simulated settings to real-world clinical practice has been limited [21,22]. In reviews of simulation-based health care education research, only 14 papers, published between 2006 and 2013, reported the transfer of learning from a simulation laboratory, resulting in improved clinical care or better patient outcomes [23,24]. It has been suggested that integrating game elements into traditional simulation-based health science learning might improve students’ knowledge, skills, and performance transfer by triggering intrinsic motivation to choose self-persistence in learning [25]. However, in a systematic review of 42 serious games used to train health care professionals, only 1 reported the transfer of learning from the educational setting resulting in improved patient care [26]. The authors’ concluded that although serious gaming in medical education continues to establish itself, more work needs to be done to define best practices for its design, development, and evaluation. More recently, Kuipers et al [27] conducted an extensive systematic review of games and simulations for health care education and found none that included a cognizant design process focused on real-world transfer. A total of 12 of the 15 studies reviewed described subconscious design features that resulted in literal transfer and 3 studies that suggested figural transfer. The authors describe literal transfer as lateral real-world application and figural transfer as the application of new knowledge and skills across different problems or situations. The authors conclude that real-world transfer is mainly mentioned as a desired outcome in research related to games and simulations for health care education, not as a guide in the design process. They suggest that a conscious design rationale is needed to optimize the real-world transfer conditions.

At present, there is no consensus in the literature regarding optimal design elements to maximize the efficacy of serious games for health care education [28]. A serious game is generally described as an interactive digital game that is designed for an educational purpose that presents a challenge that a player needs to overcome to achieve an educational win state. In addition, player feedback is provided during game play (eg, points and penalties) to enable players to monitor their progress toward a win state [29]. Some authors include entertainment as a required design element of serious games, but others do not [28,30]. Other design elements such as narrative (story), competition or collaboration, chance (random events), and levels are often debated as either required or discretionary characteristics of serious games [12,29-32].

If the aim of instruction is to ultimately transfer new knowledge, skills, attitudes, and behaviors from health care academic settings to real-world application, simulation-based or game-based learning for the health professions must have a learner-centric design to facilitate intrinsic motivation so that students self-persist to achieve mastery [5,12,13,15-17]. To capitalize on the promise of serious simulation games in health care education, it is particularly important for instructional designers to understand the underlying values, attitudes, and beliefs that might motivate a predominantly female population of students to independently choose to persistently play to mastery.

Existing gender-based game research related to commercial hardcore and casual games can help serious game designers understand why the design elements of these popular games may alienate many female health science students. Hardcore video games are targeted for and predominantly consumed by males. Typical hardcore games such as *Grand Theft Auto* feature aggressive, competitive, and violent male protagonists who seek to achieve nonforgiving, high-stakes win states, often surrounded by hypersexualized secondary female characters who are either victims or damsels in distress [1,6,15,16,33-36]. In contrast to hardcore video games, casual video games such as *Diner Dash* and *FarmVille* are designed for and consumed predominantly by females. Typical casual games feature achieving forgiving low-stakes objectives in collaborative social settings and involve a manageable investment of small chunks of time in games that are easy to learn [1,35,37]. The stereotypical male/female binary design of such games should be avoided in the development of serious games for any prospective player whose motivation to play games may be much more nuanced [16,37]. Progressive and innovative serious game design should consider nonbiological masculine and feminine characteristics across race, ethnicity, socioeconomic position, nationality, and age. Designers should consider the target audiences’ physiological reaction to visual, emotional, and tactile stimuli, and the situational cognitive-social context in which students will be exposed to the game should be understood before its development [5,12,15-17]. Finally, serious game designers need to understand the important affective parasocial connections that prospective players have with media characters in television (TV), movies, music, and books to promote engagement [5,38]. It has been suggested that the design of game characters’ attributes that align with existing important affective parasocial media connections may result in greater player engagement through identification, representation, and a deeper sense of relatedness [5,38].

### Study Purpose

In this study, semistructured individual interviews were conducted with 12th-grade female health science students who self-identified themselves as not playing games at all, not very often, or infrequently in a prior study by the authors [9]. It was expected that linking the existing gender-based gaming research with the results of these interviews might add a more richly detailed understanding that would inform the design and development of a serious simulation-based game that nongaming female emerging health care preprofessionals would be motivated to play. Specifically, the aim of this study was to seek answer to the 2 following questions:

- What values, attitudes, and beliefs contribute to the nongaming behaviors of 12th-grade female emerging health care preprofessionals?
- How do the values, attitudes, and beliefs of 12th-grade female emerging health care preprofessionals align to important design features of serious games?

## Methods

A qualitative sample study design was employed using values and descriptive coding methodology. Values coding is appropriate for studies that seek to explore the values, beliefs, identity, and interpersonal and intrapersonal experiences of purposefully selected participants [39,40]. Semistructured interviews (Multimedia Appendix 1) were conducted with 8 12th-grade female health science students; 2, 5, and 1 of whom reported playing games as *not at all*, *not very often*, or *1 to 2 times per week*, respectively.

### Sample and Participants

A purposeful sampling approach was used to identify 33 12th-grade female health science students who reported playing games as *not at all*, *not very often*, or *1 to 2 times per week*. A total of 12 young women, representing 6 racially and economically diverse northeastern US high schools, were asked to participate. Overall, 8 students volunteered to take part in the study (Table 1). These interviewees represented a diverse subset of 37 college-bound female STEM students who were dually enrolled in high school and a competitive emerging health professional (EHP) career and technical education (CTE) program. Each of these students had completed the prerequisite coursework for biology, chemistry, and trigonometry and had grade point averages of 3.0 or better. These students spent 1.5 days per week in health science–related coursework at a CTE-affiliated local state university or community college and 1 day per week shadowing health professionals at various local hospital campuses [41]. Two of these students plan to be surgeons after completing their biology/premed undergraduate degrees. Three were enrolled in 5-year accelerated physician assistant programs. One student was enrolled at a university with a guaranteed medical school track; she wants to become a pediatrician. Another student aspiring to be a pediatrician plans to complete her undergraduate degree in health policy administration before enrolling in a medical school as she wants to open her own clinic someday and needs both business and medical knowledge to do so. Finally, 1 student was still undecided regarding the type of physician she wanted to be. “I think I’ll figure that out,” she said, “as I’m going through my undergraduate degree” as a biology/premed major.

Written consents from both parents and students were obtained for these interviews, and the study was approved by the institutional review board of Lehigh University. The study adhered to the Consolidated Criteria for Reporting Qualitative Research guidelines [42].

**Table 1:** Participant profile.

Student	Race	Weekly computer time	Weekly game time	High school demographics, n (%)				Declared undergraduate major
				White	Hispanic	Black	Asian	
Harper	White	Every day	Not very often	807 (57.81)	382 (27.36)	125 (8.95)	82 (5.70)	Biology/premed
Kim	Black	Every day	Not very often	807 (57.81)	382 (27.36)	125 (8.95)	82 (5.70)	Health policy administration
Carrie	White	Every day	Not very often	2095 (79.99)	235 (8.97)	104 (3.97)	157 (5.99)	5-year physician assistant
April	White	Every day	Not very often	914 (88.91)	44 (4.28)	16 (1.56)	28 (2.72)	5-year physician assistant
Emma	White	Every day	Not very often	687 (91.97)	37 (4.95)	8 (1.07)	15 (2.01)	Biology/premed
Aria	White	Every day	Not at all	2095 (79.99)	235 (8.97)	104 (3.97)	157 (5.99)	Biology/premed
Olivia	Asian	Every day	Not at all	2308 (72.97)	379 (11.98)	94 (2.97)	316 (9.99)	Premed
Mia	White	Every day	1-2 times per week	313 (11.99)	1775 (67.98)	496 (19.00)	26 (1)	5-year physician assistant

### Data Source and Procedure

Semistructured interviews were conducted with 8 students between April 1 and April 29, 2019. Twelve predetermined self-conceptualization questions were used related to the students’ interests; values; and engagement in academic, athletic, extracurricular, volunteer, and leisure activities (Multimedia Appendix 1). Follow-up questions were asked based on the student’s responses to the predetermined questions related to associated game design elements such as competition, collaboration, feedback, and personal win state. The duration of interviews ranged between 18 min 35 seconds and 30 min 26 seconds (mean 22 min 37 seconds). The data from transcribed audio recordings of the interviews were systematically coded using the data analysis software, NVivo Plus 12.

### Data Analysis

The data analysis process was rigorous, iterative, and occurred concurrently throughout the study, starting with interview transcriptions [39,43]. Specifically, data coding occurred in 2 cycles [40]. The first cycle analysis distilled the data into core topical units using deductive and inductive values and descriptive coding as they emerged during the interviews and transcriptions [40]. New data were compared with previously coded data, with codes added, modified, and/or eliminated as data were analyzed. A blend of 45 deductive and inductive value and descriptive codes were used for the first cycle coding. A total of 14 deductive value codes were used based upon the expectancy-value motivational theory [44], the theory of expertise and expert performance [45], and the self -determination theory [46]; 21 deductive descriptive codes were based upon those used by other simulation-based learning and serious games researchers [19,25,31,32,47-53]; and 10 additional inductive descriptive codes emerged during the transcription. The codes were eliminated, modified, or recoded to fundamentally similar codes during the first cycle analysis, which resulted in 17 final codes (Table 2). The first cycle coding definitions and illustrative quotations from interviewees can be found in Multimedia Appendix 2.

The second cycle of analysis used axial coding, which synthesized the data from the first cycle coding into 4 broader, more encompassing categories. These categories were further synthesized into 2 dominant themes [40]. The student interviews, coding, and analysis were conducted by the first author. Coding in most small qualitative studies is typically a pragmatic *solitary act* [40] by a single researcher. The first author has been teaching health professionals for 38 years and is a doctoral candidate with training and interview experience in a research-intensive teaching, learning, and technology PhD program. The second author audited the coding and analysis to ensure methodological coherence.

**Table 2:** Deductive and inductive codes.

First cycle codes	Interviewees, n	Responses, n	Second cycle codes	Themes
Games	8	52	Return on invested time	Accomplishment
Work ethic	8	34	Return on invested time	Accomplishment
Time constraints	6	11	Return on invested time	Accomplishment
Cost value	7	34	Return on invested time	Accomplishment
Games	8	52	Achievement orientation	Accomplishment
Feedback	8	23	Achievement orientation	Accomplishment
Concrete actionable challenges	4	11	Achievement orientation	Accomplishment
Purposeful practice	6	7	Achievement orientation	Accomplishment
Competition	8	23	Achievement orientation	Accomplishment
Mastery success measures	4	10	Achievement orientation	Accomplishment
Physical fidelity	5	15	Achievement orientation	Accomplishment
Games	8	52	Social orientation	Affiliation
Family/community	8	27	Social orientation	Affiliation
Beneficence	7	22	Social orientation	Affiliation
Leadership	5	10	Social orientation	Affiliation
Games	8	52	Relevance	Affiliation
Introspection	8	34	Relevance	Affiliation
Smartest girl in the room	5	9	Relevance	Affiliation
Personally meaningful stories	8	40	Relevance	Affiliation
Confidence	4	6	Relevance	Affiliation

## Results

The findings suggest that the study participants exhibit a complex fusion of desire for both *accomplishment* and *affiliation.* They consciously self-regulate their active engagement with the academic, athletic, extracurricular, volunteer, and leisure activities they value. These valued activities are compatible with present or long-term goals that are sequentially reinforced in socially relevant contexts. These findings are aligned with the self-determination theory [46], which is grounded in the assumption that students are intrinsically motivated to seek out challenges and productive activities that extend their existing capabilities. These students expressed internally regulated thinking and behaviors that prioritized their sociocognitive and affective resources based upon activities that foster autonomy, competency, and relatedness.

The findings also support those of other researchers who suggest that targeted female learners cannot be simplistically defined by serious game designers as biological females with stereotypical feminine attributes [5,15,37]. These students articulated a multifaceted mixture of commonly prescribed masculine and feminine characteristics. The masculine characteristics expressed indicated that all students were fiercely independent and competitive leaders. They embraced overcoming expected failures; thought strategically; and voiced tactical, self-regulated, time-investment behaviors to achieve personally meaningful, nonforgiving, high-stakes future win states. These female students also expressed feminine gender attributes such as empathy and compassion. Prosocial thinking, attitudes, and behaviors were articulated throughout all interviews related to interactions with peers, teachers, coaches, families, and communities. In addition, the parasocial connections that these students have with personally meaningful fictional media personas, synthesized with their mixed gender characteristics, provided a compelling depiction regarding how these students self-conceptualize their present and future selves. Ryan and Deci [46] suggest that student identification, and the need to be socially connected to important others, leads to a more self-determined and autonomous form of motivation that is centrally important for learning activities to be internalized.

Collectively, the overarching themes of *accomplishment* and *affiliation* provide evidence regarding the values, attitudes, and beliefs that contributed to the nongaming behaviors of these female emerging health care preprofessionals. Conversely, these values, attitudes, and beliefs can also be used to inform the design of a serious health science game that these students will be intrinsically motivated to play. These findings support those of Jenson and de Castell [10], Kneer et al [15], and Shaw [5,17] who suggest that a blend of self-conceptualized identity characteristics might be a better predictor of a player’s choice to exert focused effort in game immersion than biological sex.

### Accomplishment

The theme of *accomplishment* was synthesized through the strongly associated subthemes of *achievement orientation* and *return on invested time*. These students devote whatever time is necessary to personally relevant, difficult, practical, and highly competitive endeavors in pursuit of long-term goals while consciously avoiding what they consider to be non–goal-oriented activities, such as playing hardcore, casual, or online video games. Beyond the number 1 ranked priority of *schoolwork first*, which was expressed by all 8 students, 6 of these students play high school varsity or club sports, and 3 work between 3 and 5 days per week at part time jobs. Regardless of their perception of games as either an appealing or unappealing leisure activity, their active decision-making processes to make the most responsible return on invested time choices were readily transparent in all interviews. One unexpected finding was that the current commercial gaming activities reported by some of these young women on the demographic survey were not consistent with their gaming activities as younger children reported during the interviews. This reprioritization of playing games as a leisure activity between the ages of 18 and 24 years has been reported by others. In their study of 190 female undergraduates, Winn and Heeter [54] reported that the student’s lack of available discretionary time was the primary reason for rarely or never playing games.

When she was younger, Harper played *Call of Duty* with her brother. She also used to play surgery games on her iPad all the time but does not play games very often now as she is so busy with the EHP program which “puts [me] ahead of other kids [my] age.” April, Carrie, and Emma, who also reported playing games *not very often*, prefer card games now to video games. A triple jumper for her varsity track team, April, used to play *Mario Kart* with her sister when they were younger, but “then just kind of like didn’t anymore. I don’t know,” she says, “It just kind of stopped.” April likes to play a variety of card games such as *Poker* and *Spit*, “and it gets [way] too competitive,” she said. The responses from Carrie and Emma were similar. Carrie reported that she used to play *Wii* when she was “little” but is no longer interested in video games. She too enjoys playing card games such as *Hand and Foot*, *Spit*, and *Golf* with family and friends during the very limited time she devotes to leisure activities. “I do try to relax or hang out with my mom or friends,” Carrie said, “for at least 2 hours on a Friday or Saturday.” When asked why she did not play immersive multiplayer games with her devoted gamer boyfriend, she air-mimicked fingering a controller and rolled her eyes while saying:

I wouldn't say there was...isn't a like a particular reason. I just feel...maybe that I would just...I don't want to spend all my time like...like watching a screen. I'd rather like get...I'd rather like go outside or something like that.

A varsity soccer player, Emma, also works as a part time waitress 5 days a week anticipating the need for spending money in college. She has liked some video games such as *Mario Kart* in the past, but now also prefers card games such as *Uno*, “because I’m super good at it.” Emma reported not playing video games “like the boys play on like the Xbox.” She said, “I do not like those...I don’t even like watching them.” Emma described the cost-value struggle of making harder future choices saying, “Hanging out with my friends,” she said, “is obviously the thing I want to do most...but it’s kind of hard...when people ask me to hang out and I have to say no because I have work or have to study for an exam.” Olivia, who reported not playing any video games at all, admitted to doing so on occasion if she is really bored in class. “I just like open up *Fire Boy and Water Girl*,” she said, “but that’s like too rare to write down that I play games.” Olivia actively participates in other high-stakes competitive gaming endeavors such as varsity volleyball, where she is currently ranked 17th in conference “kills,” as well as the Science Olympiads. Aria confirmed that she “never plays games—not even on my phone” as “it would take up all my time” and “it’s not good for me.” A varsity soccer player, Aria, also works part-time as a waitress. She also described the cost-value struggle of making present choices to achieve long-term goals saying, “it’s kind of hard sometimes...like the juggling...you know...when I feel like having fun but I have to do something else...that’s difficult for me...for sure.” Kim, who reported playing games *not very often*, was an avid gamer when she was younger, citing games such as *Wii Athlete, Just Dance, Mario Kart,* and *Nick Junior*, which she played frequently. A varsity track record holder, Kim, said, “I just don’t have time” to play games, emphasizing, “I don’t even read anymore...like that says a lot.” Describing her preferred leisure activity, Kim said:

I loved reading ever since I was little. I would [come home from] the library with like 10, 15, 20 books and I would be stuck in my room. It got to the point when my mom had to hide my books and tell me to come out because I would literally just read book after book after book and not get tired of it.

Given her current academic and athletic priorities, Kim limits her game play to periodic, brief 15-min rounds of *Temple Run* on her phone or playing *Just Dance* or something on the *Wii* “if there’s a family thing happening or kids from my church come over...but that rarely happens.” Finally, Mia, who reported playing games 1 to 2 times per week, used to play games such as *SimCity* a lot but plays less now because of her busy schedule. A varsity field hockey player, Mia, works a 4-hour shift as a waitress 5 days a week to pay for car insurance, gas, and repairs, in addition to money for college. Although Mia too chooses reading as her preferred leisure activity, she still does play *SimCity* or *BitLife* to relax periodically, but concurrently admits that it “wastes my time.”

All 8 students in this study expressed a bias for excellence in all academic, athletic, extracurricular, and volunteer activities, where they chose to invest their time. They reported embracing concrete actionable challenges, expected needing to overcome failure to achieve their goals, and appreciated varying degrees of feedback while practicing to improve incremental performances. The type of feedback that was considered motivational, however, was situational and varied among the students interviewed. When discussing when she played a lot of surgery games on her iPad, Harper shared that she does not like to be fed all the information needed to solve a problem, saying:

I like to figure it out for myself. A helpful clue here or there is nice...if you’re really stuck...[but]...you have to make mistakes to learn...actually learn.

Carrie echoed this characteristic of independently solving academic challenges herself and only goes to her teacher for helpful suggestions if stuck. April described herself as a visual learner who needed more specific feedback using an example of how watching videotapes helped improve her long jump performance. April’s coach used the videotapes to show her what she was doing wrong. “This helped me,” she said to see “what I look like” compared with “what it should look like” so “then I can fix it.” Conversely, Olivia sometimes likes feedback that is a bit more intense. “I like when people yell at me,” Olivia said. “I improved [in Volleyball] the most my sophomore year,” with a more clamorous coach, she said. “We would stand on these boxes and then I’d be hitting and [my coach] would be yelling...like harder...come on...harder...hit to this spot...and [if] I missed she’d be like...come on...why did you miss it?” Similarly, Kim expressed a need for more demanding feedback. “My coach,” said Kim:

he always pushed me...and I could take that from him. He would push me...and I’d say, ‘Coach, I can’t’, and he’s like, ‘listen, you’re fine.’

This type of tough, unforgiving feedback was intimidating for Aria and Emma who stated a preference for a softer, more encouraging approach. “We always played better [soccer],” Aria said, “with the assistant coach who let us have fun…but at the same time...like showed you how to be like the best player you can be.” Emma echoed these feelings saying, “I like someone who’s able to like tell me what to do...and like respects me as well.” Mia described a balance, expressing that the degree of feedback needs to be commensurate with performance expectations. “Our [Field Hockey] coach didn’t really discipline us,” she said. “I thought that if she did, we would have been better in shape...and our season probably would have turned out a lot better if there was more discipline.” The team, she said, “just joked around...we weren’t really serious about it anymore.” In addition, Mia imagined that “we would have respected [the coach] a little bit more” had there been more disciple.

### Affiliation

The overarching theme of *affiliation* was synthesized through the strongly associated subthemes of *relevance* and *social orientation*. All 8 young women expressed important relationships with others when describing their academic, athletic, extracurricular, and leisure activities. Their thinking and attitudes about relatedness clearly shaped how these students conceptualized their present and projected future selves. The students’ identification with others was influenced by real-world peers, family members, coaches, and mentors. All 8 students valued relevant and personally meaningful shadowing experiences with health professionals at various local area hospital campuses, which validated or helped to reconstruct their future aspirational selves [55]. Five of these students chose to invest their limited leisure time in empathetic prosocial volunteer activities, and 4 were in academic and/or voluntary leadership positions. These students did not perceive themselves to be unimportant or powerless in these roles and often emerged as active, confident leaders who achieved meaningful results. All 8 students who responded that they played games or participated in athletic activities as a leisure activity in the past or present almost always associated it with a meaningful connection to others.

Affective parasocial connections with favorite fictional characters also appear to have contributed to the students’ self-conceptualization [56]. Lucas and Sherry [3] argue that choice in media consumption to meet individual gratification is one possible source of influence in determining future choices. Shaw [5] supports this idea and has suggested that the totality of media consumption across TV, films, books, and music might offer unique insights into an individual’s self-conceptualization that can help inform designers to predict potential individual gameplay preferences. Chen et al [38] also urge designers to consider more nuanced audience analysis questions about the specific affective experiences of students as they participate in broader media consumption because girls are typically less drawn toward computer games than boys but do not show differences in their interests for movies. Relevant and personally meaningful fictional characters for this study cohort included 8 female, 2 male, and 1 animated persona. These characters were decidedly different than the hypersexualized females, damsels in distress, or stereotypical beauties that have been described in digital hardcore and casual game research to date such as Laura Croft (*Tomb Raider*), Daphne (*Dragon’s Lair*), and Kim Kardashian (*Kim Kardashian: Hollywood*). This finding, in addition to the cited, more gender-neutral, digital games played by these students, suggests that there was no alignment for these health science–oriented students to take on a compatible role of player in such games [16,30,56]. Conversely, the students’ identification with their favorite fictional characters, and their stories, was often aligned with descriptions of their cognitive, social, and affective real-world choices and relationships.

April, who is enrolled in an accelerated 5-year physician assistant program after graduation, credits a babysitter who spoke about her EHP experiences for initiating her interest in the medical field. “When I came into this [EHP program],” April said:

I was thinking that I wanted to work in the emergency room as a physician’s assistant, but I’ve had the opportunity to shadow...in the Neonatal Intensive Care Unit and Pediatric Intensive Care Units...so now I’m thinking maybe I’ll go that way or even the pediatric ER.

She identified her favorite fictional character as Hermione Granger, the overachieving muggle-born witch in the Harry Potter novels, who excels academically. Hermione is someone who does “whatever she wants to do,” April said:

[not] what everybody expects her to do. She’s kind of like making her own like path...at the end she's like taking like three classes at a time and she's popping from like class to class because that's what she needs to do. She's always like super smart like figuring out how to do everything and thinking on her feet...and she's like sort of a leader but not always like the leader.

Her description of a super smart Hermione Granger, who is just doing what she needs to do to be successful, may help validate April’s self-conceptualization and her prioritization of the unpopular, harder classes that she cares about the most, such as advanced placement chemistry and calculus. After a 5-year commitment as a counselor working at a summer camp for kids with special needs, April recently joined the junior board of directors for Camelot for Children to help increase donations. April likes playing card games with her family when she has time.

Sixteen-year-old Starr Carter, an economically disadvantaged black girl who attends an elite, primarily white boarding school in the book *The Hate U Give*, was Kim’s favorite fictional character. After witnessing a police officer shoot and kill her unarmed best friend, Starr Carter must overcome her trauma and those who seek to disempower her in her divergent socioeconomic and political realities. Kim, also a minority student, is immersed in 2 very different socioeconomic academic settings. A black female who demographically represents just 8.95% (125/1396) of her economically disadvantaged high school and 3% (2/58) of her predominantly white EHP class, Kim, admired Starr Carter’s courageous transformation into an advocate for truth and justice. She explained that her interest in medicine was influenced by her certified nurse assistant parents who challenged her to be a physician instead of the nurse practitioner she initially aspired to be. Kim, who had decided to become a pediatrician someday because of her squeamishness around blood, was animated as she explained how her enduring self-perception had been proven false when she observed an open-heart surgical procedure. “I was right next to the surgeons,” she said.

They let me come forward and see everything. I saw the entire surgery...I wasn’t squeamish...like...I was just fine.

Kim will soon represent the first generation of her family to attend college, and her undergraduate trajectory is unique. Unlike her EHP peers, Kim plans to earn her degree in health policy administration so that she understands the business aspects of running her own clinic before enrolling in medical school to become a pediatrician. When Kim spoke about varsity track, she never mentioned her record-breaking accomplishments but richly described her role as a leader within her athletic family. “When I was a freshman,” she said:

we had these two athletes...they were boyfriend and girlfriend...we called them mom and dad...they were the ones who took us in. But now that I’m the senior...like I’m taking everybody in...Like I’m taking everybody under my wing. I’m always there for them and they’re always there for me.

Kim, who described herself as a digital “gamer” when she was younger, rarely plays games now because of her academic and athletic priorities. When she does play, it is usually with family or friends.

Aria’s favorite character was Deborah Dobkins from the TV show *Drop Dead Diva*. A vapid aspiring model, Deborah’s shallow soul is brought back to life in the body of Jane Bingum, who was a brilliant, hard-working, charitable, plus-size lawyer. Deborah Dobkins is “not very smart,” Aria said.

Like...not saying that models aren’t very smart...but she wasn’t very smart...but she walks into the courtroom with like so much confidence and she just wins every time. And it’s so crazy to me...like knowing that she’s not qualified...but she can just do it.

Interestingly, Aria addressed being similarly conflicted, sometimes feeling like 2 personas in 1 body, continuously choosing between the roles of serious, responsible student and just wanting to have fun and hang out with friends. As a freshman, Aria had a good friend in the EHP program who just loved what she was doing. “She told me a lot about the program...and I was like...that sounds amazing...like that’s what I want to do,” Aria said. Since those initial conversations, “there’s never been like a question for me...like saying that I want to go into the medical field.” Aria continued, “like it’s the only thing I can see myself doing.” Aria will begin her undergraduate studies as a biology/premed major after she graduates high school, aiming to pursue medical school someday. Aria described herself as “a pretty busy gal...in like a ridiculous amount of clubs,” but she values being involved in her school. As the secretary of the Pediatric Cancer Club, she recently led a *Shave for the Brave* event that raised over US $100,000 for pediatric cancer patients. Aria also mentors newly enrolled students at her high school to help ease their transition. Aria confirmed during the interview that she does not play games as it would not be a good use of her time.

A white female who demographically represents just 11.99% (313/2611) of her economically disadvantaged high school, Mia, has been accepted into an accelerated 5-year physician assistant program. She explained that she began “wanting to find her spot in the healthcare field” as she had a good friend who was diagnosed with leukemia when they were very young, and the people who cared for him motivated her interest. Mia’s favorite fictional character was Thomas Edison, the amnesic protagonist in the novel *Maze Runner*, who transforms himself from a scared, confused adolescent to a courageous, decisive leader in a dangerous apocalyptic world. “I like how he just like took over everything,” Mia said. “He was different than the others [in the maze]...he wanted to lead them to safety and no one else did...they didn’t want to go...they didn’t want to do anything.” Mia may identify with Thomas’ initiative to escape his maze as she too is different academically and planning to escape her own low-achieving high school “maze,” which is ranked 516 of 673 schools in the state, with math proficiency and reading proficiency scores of 32% and 43%, respectively. When one considers Mia’s feelings regarding her new CTE peer group compared with the demographic environment of her high school, her identification with Thomas seems even more salient. “I’ve created a better relationship with my friends here,” Mia said. “All the kids here are interested in the medical field...I had people that related to me...and understood what I was going through...like picking colleges.” Mia was the most frequent game player of this group of students, who reported playing *SimCity* “all the time” when she was young and still plays 1 to 2 times per week when she is bored or needs time to relax. In the *SimCity* games, players build their own societal stories to fit their own desired cultural contexts [1,5,16]. Given Mia’s low achievement and economically disadvantaged high school environment, her description of the *SimCity* character she created is noteworthy. “Right now, my girl...I made her,” Mia said. “She lives in the city...and I took her to college for a business degree...and then she spent like three weeks there... and now she’s in Paris on vacation.” As the president of Key Club, Mia increased the annual benevolent Key Club projects by over 400% by adding community food drives and monthly parent-teacher association meeting babysitting services. When Mia entered the EHP program, she wanted to be a pediatrician but decided against becoming a doctor while shadowing them because of conflicting lifestyle choices. Mia did not like seeing physicians who were content working while their families were on vacation. “That wasn’t me...that was not me at all,” she said. “I want to have a life. I want to travel and have a family.”

## Discussion

A more nuanced analysis of prospective female players has been advocated before the design and development of serious games. This study sought to better understand the values, attitudes, and beliefs that contributed to the nongaming behaviors of 8 12th-grade female emerging health care preprofessionals. It was anticipated that these understandings would help inform the design of a serious game that these students might be intrinsically motivated to play.

### Principal Findings

The findings of this study suggest that these 12th-grade health science–oriented students exhibit a complex fusion of desire for both *accomplishment* and *affiliation* representing a multifaceted mixture of commonly prescribed masculine and feminine characteristics. These young women are all are fiercely independent, introspectively competitive, prosocial leaders who are intrinsically driven to pursue health care careers. They think strategically and have tactically structured their past and present lives to achieve personally meaningful long-term goals. They embrace overcoming expected failures to achieve relevant high-stakes wins in all academic, athletic, extracurricular, and volunteer activities they value. They consciously avoid what they consider to be non–goal-oriented activities and subsequently self-limit their leisure time. When investing constrained leisure time, they choose to do so in socially meaningful contexts, which may include rarely playing games. On the basis of these common student attributes, the following design elements are recommended to foster autonomy, competency, and relatedness when seeking to motivate nongaming female students to independently choose to persistently play a health science–related serious game to mastery.

### Implications for the Design of Serious Games

With regard to the theme of *affiliation,* it is expected that these preprofessional health care students will be more motivated to engage in a serious game that contains a relevant story [47,57]. Emotionally placing these students in a game environment with near real-world patient characters should trigger their feelings of empathy and compassion. Engagement with such characters in a meaningful and emotionally rich story may lead female health science students to identify with patient personas resulting in inherent prosocial behaviors and feelings of responsibility and accountability [12-30]. These relevant stories should unfold in an authentic clinical practice environment, which combines near real-world social interactivity and authentic behavioral dynamics and likely workflow distractions [19,32,48,51]. It has been suggested that eliciting emotional arousal in learners may result in the student’s self-regulation and motivation for achievement [58-60]. It is expected that well-designed emotional triggers provided by nonplayer patient characters in authentic clinical environments will align with the existing values, attitudes, and beliefs of female health science students. The resulting interactive parasocial relationship between these students and nonplayer patient characters should impact the active choices these students make to persist to achieve in the face of whatever concrete actionable challenges they face in the game.

With regard to the theme of *accomplishment,* authentic, concrete, actionable challenges should be designed into the serious game, which improve the fictional patient’s clinical status while protecting them from harm [19,32,51]. It is expected that female health science students who identify with nonplayer patient characters will embrace solving their clinical problems by learning to use, manipulate, and reconfigure applicable tools while navigating visually rich authentic clinical environments [8]. Increasingly difficult levels of incremental knowledge, skills, attitudes, or behaviors should be achieved through repetitive, increasingly difficult practice until the students reach an individually meaningful win state in which their fictional patient’s clinical issues are resolved without any adverse events [19,32,47,51]. The serious gaming environment should provide these students with nonforgiving, high-stakes feedback in the form of performance-based rewards and performance-based penalties, which are aligned with incremental successful or unsuccessful fictional patient care based upon knowledge, skills, attitudinal, or behavioral benchmarks that are clearly defined and measured [19,25,32,47,49,51]. Finally, the design of instruction should allow each student to decide how he or she chooses to respond to likely workflow distractions, or time constraints, to achieve success at each instructional level [8,19,32,49,51].

### Limitations

The participants in this study represented 18.6% of the total female EHP student population and 21.6% of the original study cohort in prior research reported by the authors [9] and reflect a small sample size. The themes that emerged in this study may not reflect those of the collective group of college-bound health science learners in this class or those of incoming classes.

### Conclusions

The results of this study reinforce the need for a robust learner analysis to identify the multifaceted behavioral characteristics of targeted learners before the design and development of serious games. The common characteristics of the 12th-grade female health science students in this study indicate that they will choose to invest their limited leisure time playing a serious game if it is aligned with the self-conceptualization of their present or future selves. It is expected that these students will choose to solve sequentially difficult concrete, actionable, patient care challenges if they parasocially identify with the nonplayer patient characters in a relevant story that takes place in an authentic clinical practice environment with authentic behavioral dynamics and likely workflow distractions. It is expected that female health science students will choose to persist to an individually meaningful win state on behalf of their nonplayer patient characters as they receive formative feedback in the form of performance-based rewards and performance-based penalties, which are aligned with incremental successful or unsuccessful procedural care.

## Appendix

Multimedia Appendix 1 Semistructured interview guide to better understand the motivational dynamics of why 12th-grade emerging health professional students might choose to play games.

Multimedia Appendix 2 First cycle coding definitions and illustrative quotations from interviewees.

## Abbreviations

CTE: career and technical education
EHP: emerging health professional
STEM: science, technology, engineering, and mathematics
TV: television
