# Supplementary material for: Learner Analysis to Inform the Design and Development of a Serious Game for Nongaming Female Emerging Health Care Preprofessionals: Qualitative Sample Study
Source: JMIR Serious Games. 2020 Feb 6;8(1):e16003. doi: 10.2196/16003 (PMC7055850; doi:10.2196/16003)
Supplement: Multimedia Appendix 1 [file games_v8i1e16003_app1.pdf]

Multimedia Appendix 1

Semi-Structured Interview Guide to Better Understand the Motivational Dynamics of Why 12th Grade Emerging Health Professional Students Might Choose to Play Games.

Predetermined questions (1-12) will be balanced with emergent questions related to game mechanics* that arise in response to participant's shared thoughts.

1. Who is your favorite fictional character? Why?
   (*e.g. meaningful story, concrete actionable challenges, social engagement)
2. Why did you apply to the EHP program?
   (*e.g. meaningful story, personal win state, beneficence, collaboration)
3. What kind of healthcare professional do you think you want to be? Why?
   (*e.g. relevant story, physical interactivity, behavioral interactivity)
4. What was the most memorable thing about your EHP Program so far?
   (*e.g. physical interactivity, purposeful practice, behavioral interactivity)
5. Do you participate in any activities after school – sports, clubs, job, etc.?
   (* e.g. personal win state, concrete actionable challenges)
   a. What do you like most about it?
      (*e.g. competition, collaboration, purposeful practice, success measures)
   b. What do you like least about it?
      (* e.g. competition, collaboration, purposeful practice, success measures)
6. What are your plans after you graduate high school?
   (* e,g. personal win state)
7. What is the one thing do you enjoy doing the most every day? Every week?
   (* e,g. personal win state, behavioral interactivity)
8. Do you ever play games? Why? Why not?
   (* e,g. personal win state, time constraints, behavioral interactivity, competition)
9. What game do you play most regularly? Why?
   (* e.g. collaboration, random events, behavioral interactivity, concrete actionable challenges)
   a. What do you like most about it?
      (e,g. collaboration, random events, behavioral interactivity)
   b. What do you like least about it?
      (e.g. competition, time constraints, random events)
10. If you could pick any secret alter-ego, who or what would it be? Why?
    (* e.g. personal win state, success measures, meaningful stories)
11. How do you like to be taught, coached, supervised?
    (* e.g. feedback via physical interactivity, purposeful practice and/or behavioral interactivity)
12. When you have a crazy busy day, what are the most important things you prioritize?
    (* e.g. personal win state, time constraints)
    a. What things do you sacrifice to get the most important things done? Why?

* Emergent Game Mechanics Questions Based Upon 1-12 Predetermined Question Responses

Relevant Stories	Personal Win State	Meaningful Stories

Multimedia Appendix 1

Physical Fidelity (Authenticity-Look Real)	Concrete Actionable Challenges (Authenticity)	Psychological Fidelity (Authenticity-Feel Real)
Physical Interactivity	Purposeful Practice	Behavioral Interactivity
Random Events	Competition	Competition
Time Constraints	Collaboration	Collaboration
	Success Measures (Mastery)	Beneficence
Feedback	Feedback	Feedback
