# Supplementary material for: Learner Analysis to Inform the Design and Development of a Serious Game for Nongaming Female Emerging Health Care Preprofessionals: Qualitative Sample Study
Source: JMIR Serious Games. 2020 Feb 6;8(1):e16003. doi: 10.2196/16003 (PMC7055850; doi:10.2196/16003)
Supplement: Multimedia Appendix 2 [file games_v8i1e16003_app2.pdf]

Multimedia Appendix 2

First cycle coding definitions and interviewee illustrative quotations		
First Cycle Codes	Definitions	Illustrative Quotations
Games	Students describe a form of interactive play in which a challenge needs to be overcome to achieve a win state. Design elements include feedback (e.g. points and penalties) to enable players to monitor their progress toward a win state. Other design elements may include a theme, narrative (story), competition, collaboration, chance (random events), time, and resources (assets).	"I used to play a lot of video games [like *Call of Duty*] with my brother, but…like this year has just been so busy, so I haven't played like any games…really…I'll play [*Clue*] with my grandma on the weekends if we have time." (Harper)
		"…video games…like not really…like sometimes I'll have like a random game on my phone that I'm like playing when I'm bored but for the most part not really. I'll play card games and stuff with like family. We have like a bunch of family and friends and when all the kids are together, we often end up playing card games [like *Poker*]. (April)
		"I don't really like electronic games…I used to play the Wii when I was little…and now I just don't… I actually like playing cards [like *Hand & Foot*] with my family, and board games as well" (Carrie)
		"I used to play *Jetpack Joyride*… a course that you go through [With the jet pack on] so it feels like your flying. Once in a while I'll play…like if I'm really bored in class… *Fire Boy and Water Girl* [on my phone], but that's like too rare to write down that I play games." (Olivia)
		"I used to play *Nick Junior* when I was little. Oh, my goodness, I always played *Nick Junior.* But yeah, those are the games that I played when I was younger, but as I got older it's like…I don't have the time…if there's like a family thing happening or like if there's kids from my church that come over we just play like *Just Dance* or something on the Wii, but that rarely happens." (Kim)
		"I'm not sure why [games have] never been like a thing for me. Some of my friends will have games on their phones. But like if I had any on my phone, I just got bored of it so quickly and [it's] like…why am I even doing this…or I'll get too into it and then it's just like taking all of my time up…so that's why I just stay away from [games] because I know it's not good for me." (Aria)
		"On my computer…when I was younger, I used to play *Sims 3* a lot…like all the time I'd play that game…and that's where like you make your own person, and you move them in a house…like you control their life kind of. And so, my boyfriend is really into computers and like he found a way to get all of the *Sims 3* games for free and put them on my laptop. So, that's what he did. So, now I have that…and that…wastes my time. So, that's been a new relaxing kind of thing." (Mia)

Multimedia Appendix 2

		I do not play video games. I'm thinking about like the ones like the boys play on like the Xbox and like all those types. I do not like those. I don't even like watching them. I love card games. I love Uno and Rummy and all of those…I like Solitaire, too. Card games, yes. Board games, yes. with friends and stuff." (Emma)
Work Ethic	Students describe being industrious, organized, and highly productive	"I decided I wanted to apply for [the] emerging health [program in middle school]…so like I've known for like a really long time… there's only so many science classes that you can take [in high school] that are like medically based…like AP Bio. So, [the EHP program] was an opportunity for me to take classes that like actually pertain to what I'm going to do in the future. So… I designed my [high school course] schedule… doubling up some years so that… I didn't have to cut out any [high school] classes [to participate in the EHP program." (April)<br><br>"I work at a Mexican restaurant… I work during the week and on the weekends. I'd say on average. I work about two days a week…. usually Tuesday and Thursday and then all weekend like, Friday, Saturday, and Sunday**,** like my mom hates it…because she thinks it's too much. I do have to pay for like all my books and stuff for college and that's a lot of money. I always feel like I have to be doing something and I have an issue where if I'm not doing something that I'm doing nothing." (Aria)
Time Constraints	Students describe how they manage limited time periods to complete a task or activity	"If it's like a super busy day, it's like school work first, and then usually, it's like the harder classes are like the ones that I like care more about…like AP chem or like Calculus…like those ones are gonna like take more time and they're harder. So, I do those ones first and then English [and] social studies….just because I know I can do less [in] them and still do just as well as I would in other classes so I know that I should put more time in the ones that are a little bit more challenging." (April)<br><br>"I mean…if it's like…if it's important for my grade, I'll always put that first, but if I have the option of doing it another day, I would just do what I can get done first, easier, shorter." (Olivia)
Cost Value	Students describe where and how they invest their time and effort in an activity, and what they are willing to sacrifice to do so.	"My boyfriend plays all those shooting games …I would just rather watch TV or hang out with my friends…I don't want to spend all my time like…like watching a screen. I'd rather like go outside or something like that." (Carrie)<br><br>"I always prioritize…like school so, like if I have so much homework, I'll push off like watching TV…or hanging out with friends." (Harper)

Multimedia Appendix 2

Feedback	Students describe how feedback helps or hinders their modification of efforts to incremenally improve performances.	“One of the refs that coached our [Field Hockey] summer league tried to recruit a whole bunch of us [to a club team] …me and like three girls went to some of their practices. I didn't really like it…they split us up between people that were good and people that weren't good… I kind of felt pointed out…it kind of put me down a little bit. [The coach] was kind of just, like here, play like the good girls. He wasn't really teaching us anything…we didn't get his full attention…so I stopped going” (Mia)<br><br>“My coach freshman year actually left our team last year. So, I had a new coach for sprinting…he's really lenient…It's like if you try your best…you try your best…but like my [freshman] coach…like he's still my coach…he's back now to help with the mid distance runners. I still say like he's my coach…like because he always pushed me…and I could take that from him…like he would push me, he would push me…like case in point he always said ‘you have so much potential….you don't understand’… like he would always like…he like…when I say push…he would push me to…like to…and, I’d say, ‘Coach, I can't.’ And he's like ‘listen, you're fine. You'll be okay.’” (Kim)
Concrete Actionable Challenges	Students describe activities that push them to complete tasks beyond their current capabilities.	I used to like download like surgery games on my iPad…as many as there were**…**just like all different kinds of things…like plastic surgery or like general surgery…and some of them I didn't like so much because [the game] was kind of like telling [me] everything [I] had to do. I like to figure it out for myself… like a helpful clue here and there is nice…if you're really stuck then you can like click on that for help, but [I liked] actually performing the [surgery] and solving the issue.” (Harper)<br><br>“When I was younger… I used to play this exciting game…it was a surgery game and you were timed. You had to use the right tools. If you didn't use the right tools…time [was] taken off and stuff like that. It was stressful [and it] really made you think of which instruments to used.” (Kim)
Purposeful Practice	Students describe repeatedly practicing an activity that requires concious and focused effort to achieve a performance goal.	“We had this one coach my sophomore year of Club Volleyball and she would just yell at me all the time…sort of like incorporating an encouraging way into the yelling. So, we would stand on these boxes and then I'd be hitting and then she would just be yelling like harder…come on…harder…hit to this spot…and [if] I missed, she'd be like, come on…like why did you miss it? And that's the year that I like…improved the most.” (Olivia)<br><br>“I had been going to ballet school since seventh or eighth grade…doing two performances a year, but there was a burnout factor at the end [11th grade]. I was kind of bored…because I'm used to dancing like three, four times a week…sometimes five. now I'm running track for my school because…why not…I was like, yeah sure…like why not…like I can jump.” (April)

Multimedia Appendix 2

Competition	Students describe an intrapersonal or interpersonal activity with a defined win state.	I love Uno… because I'm super good at it… I'm very competitive." (Emma)<br><br>"I really like shadowing [health professionals in the EHP program]. Like you really do learn a lot more and it puts you ahead of other kids your age." (Harper)<br><br>"I play soccer at [my] high school. I've played since I was a freshman, but overall, it's been like 15 years…like micro-soccer all the way up." (Aria)
Mastery Success Measures	Students describe how they self-monitor and modify their own behaviors to achieve performance excellence.	"I…like hands on solving… trying to solve the problem myself and then if I need help…like going to the teacher and just talking through things**.** I also just take a lot of notes and then just rewrite them and then figure everything out…because I think…when I type…I'm just like…yeah, but then if I…it takes a lot longer when I write it out, but I feel like I might know it more" (Carrie)<br><br>"You have to make mistakes to like learn and actually learn…because like if you just make a mistake in the game…and it doesn't like give you the chance to like fix it…or like later…like do it again or like something…then it's like you're just being told and you won't really process it or understand it later if you're like asked to replicate it." (Harper)
Physical Fidelity	Students describe the environmental authenticity of an experience.	"I saw [two laparoscopic] surgeries today and…which is amazing that…like at this age already, I'm able to see that. Last week I also saw an open-heart surgery. I just was like, oh my gosh, like…that's a heart. I was right above it, like literally standing like…the heart was here…I was like right here…the bed was here…and then they had this little thing and I stood on a step stool and watched like over it. They [the doctors] don't just like leave you to the side…like they like get you into it and like tell you what they're doing…like actually like teach you." (Emma)<br><br>"I saw a natural birth and I…I was just completely…like shocked and interested in the whole process because…like…you don't like just think of what's going to happen. You just know - Oh they had a baby, but you don't actually like understand it. I just thought that was the coolest thing ever. I was almost hit with her foot…in the face. Like…I was right there!" (Carrie)

Multimedia Appendix 2

Family/Community	Students describe a cohesive unit of individuals who love and support each other.	"I would say getting all the kids together that are interested in the medical field [in the EHP program]…really like I don't know how to say it…like it kind of felt homey and that I had people that related to me…we could just talk about [healthcare] together and like…get excited and talk about what we saw during shadowing or even with the anatomy classes we took. It was nice to have those people that understood what you were going through like picking colleges…picking what you wanted to do. I made a lot of new friends. And I feel like the friends I made [in the EHP program] are different than the friends I made at [my high school] …and it's like I've created a better relationship with my friends here." (Mia)<br><br>"like [track is] really about the family…I was like pretty great freshman year, but like I didn't have like that family feeling…[but] we had like these two [senior] athletes…and we called them mom and dad because like they were the ones who took us in and everything like that. But now that I'm a senior…like I'm taking everybody under my wing…like the freshman and the new ones. I guess my personality…just in general is just like… everyone wants to talk to me and everything…we form this connection and like any time…if they need me to help them with something…I'm always there for them and they're always there for me…always cheering me on…" (Kim)
Beneficence	Students describe commpassionate pro-socially oriented behaviors.	"I was the Secretary of [the Pediatric Cancer Club] …and this year we did *Shave for the Brave*. People shave their heads in like solidarity with children who have cancer and we raised over $100,000…like it was really successful. I basically put the event together…like with a few other students. I was there every day like planning…buying things…[and] promoting the event. So that was a big deal like in the beginning of the year." (Aria)<br><br>"I'm in SADD Club, which is like students against destructive decisions… …like driving drunk…Our colors are like red…so we'll have like dress red days to like…show we're there to talk to you if you need someone…like if you feel like you're being peer pressured…like the people in the red…like you can like talk to us if you need us type of thing. You can't really do too much like, you know what I mean, but we try our best." (Emma)
Leadership	Students describe guiding a group of people on a common mission.	"This is my first year being President [of Key Club] and like the past years, I noticed we would only do like one or two things [a year]. I was like, okay…so what's the point of constantly coming to these meetings and just not doing anything? So, we [added] a food drive…a canned food drive this year. We [added] volunteering at Dodd Elementary School. It was kind of like a babysitting thing. Like, you know, how they have like PTA meetings. So, the parents would bring their kids and they would have no one to watch the kids. So, we sat there, and we play games with them for about an hour and we been doing that once a month. So, we've been doing it for three months now." (Mia)<br><br>"I'm the vice president of student government…and I go to [the school] board meetings. I just go to one meeting a month and then give them updates on [my] high school and the student body. Like

		tonight…I'll be talking about all the trip opportunities that our kids have throughout the summer and throughout spring break as well." (Carrie)
Introspection	Students describe how they self-assess their own mental and emotional processes.	"I do yoga every day. I started like…like four months ago. I teach myself [by] watching videos on YouTube. I do it sometimes when I wake up…or if I like don't do it in the morning, I'll do it like at nighttime before I go to bed. It really just like calms me down…it gets me away from like…my phone…and just like gives me like a break. Do you know what I mean?" (Emma)<br>"I'll be sitting there driving and I'll be like I'm not even singing…like I'm definitely not in a good mood…like if I'm not singing. But if…I'm sitting in the car, you now…sometimes I like to dance a little bit. If I'm doing that, I know that I'm in a good mood because…I feel like it's subconscious in a way." (Aria)
Smartest Girl in the Room	Students describe themselves or others with whom they identify as smart.	"[Hermione Granger] just like…she's doing whatever she wants to do. She's not necessarily doing what everybody expects her to do and she's like…I don't know…she's kind of making her own like path…at the end she's like taking like three classes at a time and she's popping from like class to class because that's what she needs to do…and she's always like super smart…figuring out how to do everything." (April)<br>"I really like to procrastinate and…pressure study. So, I always like…the night before…I'm like, okay in four hours, I'm going to study these four chapters and I just like go through the PowerPoints and memorize it. I don't want to sound like cocky but I'm just saying I haven't gotten…like I always get above a 95." (Olivia)
Personnally Meaningful Stories	Students describe the experiences of real world or fictional personas with whom they identify.	"I had a friend that went to school with me and he had leukemia very young. So, I was always…like interested in like…what was like…his cancer? What did it mean? And I was really interested in that. And especially pediatrics…because I had a friend so young. I liked the people helping him him…it motivated me." (Mia)<br>"My grandmother was a nurse, but that's like the only person in my family who's in the healthcare field…but I've always just been interested in it, I guess…and this is going to sound stupid but *Grey's Anatomy* like really…like it like introduced me to it…even though half of it is wrong, or incorrect, or whatever. But I just really like the fact that you can get to know someone and watch them progress and help them and…like change their life and I like medicine." (Carrie)

Confidence	Students describe self-assurance of their own abilities, charteristics, and decisions.	"I'm very good at last minute. I'm very…very good…and that's a blessing and a curse because I do like to procrastinate. Even before we started our [college] classes they were like you 'can't study the night before…like you will fail. There's only a small percentage of you that can'…and I do it every time." (Aria)<br><br>"At first, I wanted to be a pediatrician and then…kind of like with the [EHP] program…I was able to pinpoint what I actually wanted to do. Following physicians and even…surgeons…it was like they had no time for the…like they said that their kids were out on vacation and like they said they would rather be in surgery…in the OR…then being with their family and I didn't like that. That wasn't me. That was not me at all. I want to travel…and I want to have a family and I thought that if I was a full-time pediatrician that I wouldn't be able to have that time for myself…and be able to take care of myself…because I have a lot of stress and anxiety and I was like, that's probably not the way to go." (Mia)
